# Assessing rare disease understanding: a novel disease readiness level framework

**DOI:** 10.1186/s13023-025-04135-y

**Published:** 2025-12-29

**Authors:** Kazuki Kitahara, Shingo Kano

**Affiliations:** Bio-Innovation Policy Unit, Department of Computational Biology and Medical Sciences, Graduate School of Frontier Sciences, The University of Tokyo, 5-1-5, Kashiwanoha, Kashiwa-shi, Chiba, 2778562 Japan

**Keywords:** Technology readiness levels, Research progress, Disease understanding, Disease readiness levels, Research and development, Rare diseases, Clinical guideline, Regulatory readiness levels

## Abstract

**Background:**

Drug development for rare diseases has hurdles against setting high priority because of the size of the market. Although many countries have incentive policies for the development of orphan drugs (drugs used against rare diseases), evaluation methods for determining the rare diseases warranting resource support have not yet been established. To promote research and development (R&D) of rare diseases and drug development, methods measuring the level of understanding of rare diseases and its comparison with that of other diseases are warranted. This study proposes a grading system for measuring simultaneously the level of understanding of rare diseases and progress in product development.

**Methods:**

Using the Technology Readiness Levels (TRL) framework developed by the National Aeronautics and Space Administration, we proposed a Disease Readiness Level (DRL) to assess the understanding of rare diseases by comparing the characteristics of existing TRL derivatives in the medical field, adding a clinical guideline in the middle stage and extending the assessment period to earlier stages than product development. Case studies with the developed framework were conducted for four rare diseases.

**Results:**

The DRL comprehensively described the four selected rare diseases, muscular dystrophy, progressive fibrodysplasia ossificans, Tangier disease, and idiopathic peripheral pulmonary artery stenosis from their disease origin in the pre-product development phase to the launch of therapeutic strategies over a longer period than previous TRL derivatives.

**Conclusions:**

This study developed a comprehensive framework for rare diseases that focuses on the disease rather than the product for assessment and covers information spanning disease discovery to drug development. The results of case studies using the framework suggest that DRL can analyze both the level of understanding of rare diseases and the progress of the product research and development (R&D), and can be used as a potential indicator for the allocation of R&D resources.

## Background

Rare diseases (RDs), which are medical conditions with a low prevalence in the general population, have an estimated prevalence of more than 7,000 diseases worldwide [1]. A universal definition of RD is yet to be established. The average prevalence threshold established by the relevant authorities in each country is 40–50 cases/100,000 people [2]. Owing to its rarity, RDs had been less likely to be targeted for therapeutic drug development by the large pharmaceutical companies, and were developed mainly by start-up pharmaceutical companies. Some drugs, which are effective in the treatment of rare, usually life-threatening, diseases, are designated ‘orphan drugs’. Incentive programs to promote the development of orphan drugs have been established in many countries, including the United States, Europe, and Japan, which are representative drug development markets [3, 4]. However, even in the United States and Europe, where therapeutic drug development is active, most RDs still lack approved treatments [5, 6]. Between January 2005 and December 2021, orphan-designated new molecular entities and biologics approved in the United States were 244, of which only 124 were approved in Japan [7]. In Europe, only 192 Orphan Medicinal Products were approved in the period 2010–2022 [8]. The rate of translation of knowledge of RD into therapy lags considerably behind the rate of knowledge generation [6]. Medical needs, which are defined as the absence of therapies, are often claimed in orphan drug applications. However, the difficulty in effectively developing drugs for RD may be related to disparities in the “generic” definition of unmet medical needs (UMNs) according to the stakeholder’s position [9]. Moreover, no method has been established for the quantification [9] and appropriate allocation of research and development (R&D) resources for rare and common diseases [10]. Although previous research has demonstrated advocacy support as a key factor for R&D investment for RDs [11, 12], and emphasized on severity and not just rarity [13–15], no assessment methods have been established for their proper evaluation. Previous studies from the patient/caregiver perspective have focused on important research topic in specific disease areas [16–18]. START, proposed by the International Rare Diseases Research Consortium Orphan Drug Development Guidebook task team, is a checklist focusing on key pillars, stakeholder mapping, available information, resources, and value profiles of target patients to consider when initiating drug development in rare diseases [19]. While the need for a specific approach to prioritize the appropriate allocation of R&D resources across multiple diseases has been stated [20, 21], no consensus exists on a specific method. The importance of monitoring tools to assess the current state of development so that research funding and policymaking efforts can be optimally aligned has been recognized not only in the medical field but also in other industrial sectors for research and innovation assessment [22].

The Technology Readiness Levels (TRL), which were proposed by the National Aeronautics and Space Administration (NASA) in the 1980s [23, 24] and used as an official innovation policy tool in the European Union [25], are a systematic metric that assess the maturity of a new technology. The TRL is a nine-level system that assesses a particular technology from its introduction to culmination and compares the advancement of different technologies [23, 24]. Owing to its abstract and streamlined definition, TRL has been widely adopted in various fields with adjustments based on expertise [26]. For instance, previous studies have demonstrated the introduction of TRL in the medical field, including veterinary medicine and drug regulation [26–28]. In the United States, the Biomedical Advanced Research and Development Authority (BARDA) uses TRL to define a common set of definitions for each product determining the progress of R&D programs for medical products such as drugs, vaccines, medical devices and assays, in order to properly manage the amount and timing of funding effectively [29]. To complement TRL, the Commercial Readiness Index (CRI) was developed by the Australian Renewable Energy Agency. CRI is a six-level scale designed to assess the commercialization risks remaining beyond TRL level 9. TRL levels 2–9 coincide with CRI levels 1–2, and CRI extends beyond TRL level 9 to illustrate residual risks after commercialization [25, 30]. On the other hand, to the best of our knowledge, no comprehensive method for evaluating research progress, including basic research prior to TRL level 1, i.e. prior to the development of a specific product, has been reported.

In the context of RDs, there exists a significant disparity in investment and development of treatment options. There was an inequity between RDs where multiple treatment options were developed and those where no investment was made, and the inequity and the complexity of the pathophysiology was reported as a challenge in R&D [31]. In Japan, the factors influencing the increase or decrease in research funding for intractable diseases have been examined mainly based on the clinical research database [32], but there has been no comprehensive analysis of basic and clinical research to examine the optimal resource allocation, not only in Japan but also in other countries. While advances in technologies, such as exome and genome sequencing, have made it easier than ever to diagnose patients with undiagnosed diseases in various countries [33–36], the information and resources necessary for the steps from new disease identification to the development of therapeutic drugs have been sporadic and fragmented as mentioned in the Rare Disease Moonshot [37]. However, there has been no attempt to successively integrate the progress of the pre-product development phase, such as basic disease research and establishment of clinical practice guidelines, and the product development phase, by TRL. In order to promote effective R&D with limited resources, it is necessary to classify comprehensive disease research maturity not only at the stage of clinical drug development, but also from the establishment of the disease concept to the clinical practice guidelines for the target RD.

Therefore, this study aimed to develop a disease readiness level (DRL) framework designed to comprehensively measure the degree of understanding of a disease and the progress of research and development of products to intervene in R&D for individual RDs, and to test feasibility of the framework through case studies.

## Methods

### Constructing the analytical framework: DRL

The TRL framework proposed by NASA consists of nine levels to assess technology maturity [23, 24]. By incorporating and extending the TRL, we developed a DRL that encompasses the steps from the discovery of a new disease, proposal and establishment of the disease concept, basic and applied research, establishment of clinical practice guidelines, and the development/regulatory approval of therapeutic drugs. The DRL assesses the progress of medical science in the target disease, not a product. Detailed definitions of DRL are provided in Table 1. The descriptions and definitions of DRL are listed in a nine-level framework applicable to each level. For simultaneously describing both the process from the establishment of the disease concept to the proposal of clinical practice guidelines and the progress of drug development as a therapeutic tool, the process was broken down by placing the proposal of tentative clinical practice guidelines at level 5. Next, at the level preceding the development of clinical practice guidelines for RDs, there was a need to accumulate evidence on diagnosis, best practices for existing treatments, and basic genetic studies [38], which were broken down into levels 1–4. Regarding R&D activities related to therapeutic measures, the confirmation of the applicability of existing drugs occurs prior to the establishment of clinical practice guidelines (level 4), while the development of new drugs is defined as an event that occurs after the establishment of clinical practice guidelines (level 6 or later) with reference to BARDA for Medical Countermeasure Products and Regulatory Readiness Levels (RRL) [28, 29]. The details of each process are described below.

**Table 1:** Disease readiness levels

Level	DRL description	DRL definition
1	New disease identification and case reports	Potential new disease reported in conference presentations and/or scientific papers (even if sporadic)
2	Proposed disease concept	Reports of disease concepts proposed in conference presentations and/or scientific papers (generally including multiple case data) Patient organizations are established (according to the number of patients and disease characteristics)
3	Disease mechanism research in basic fields	Mechanism elucidation research (basic research) is being conducted In vivo (animal models) and/or in vitro assay system established
4	Applied research for diagnosis and treatment	First clinical treatment using diagnosis/existing drug for the proposed disease
5	Establishment of clinical practice guidelines, diagnostic criteria and disease definitions	•Guidelines and diagnostic criteria published by relevant societies •Disease names listed to the ICD10 and/or ICD11 codes •Current best and/or standard practice for treatment shared through guidelines, including non-pharmacological treatments (i.e., surgical treatment, rehabilitation, transplantation, etc.) recommended in medical practice guidelines and used in actual clinical practice
6	Screening and identification of therapeutic drug candidates	Therapeutic drug candidates screened using animal disease models (including iPS disease mimic cells)
7	Pre-clinical study	Efficacy and safety in preclinical study, Consensus building for extrapolation to human
8	Clinical trial (Phases 1, 2, and 3) conducted	•Phase 1, 2 or 3 being conducted by Clinical trial.gov, Clinical Research Portal site, etc. •Orphan drug designation is obtained (if possible) •Patient organizations could support clinical trials through information sharing and networking as PPI
9	Drug approval and launch in the market	The list of approved drugs by each authority (FDA, PMDA, EMA, etc.) can be checked to confirm that the drug has been approved and marketed for the target disease
		9a: Symptomatic treatment
		9b: Disease modifying treatment
		9c: Curable treatment

DRL: Disease Readiness Levels, iPS: induced pluripotent stem, ICD: International Statistical Classification of Diseases and Related Health Problems, PPI; Patient and Public Involvement

**Fig. 1:**
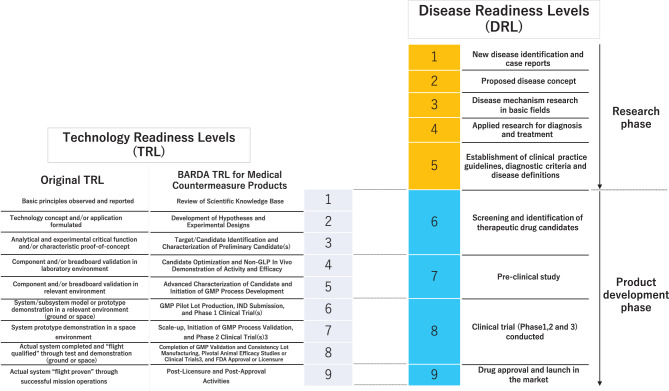
Disease readiness levels and technology readiness levels. The relationship between DRL and TRL. Levels 6–9 of the DRL correspond to TRLs 1–9. DRL, disease readiness Levels; TRL, technology readiness Levels; GLP, good laboratory Practice; GMP, good manufacturing practice

The initial phase of conceptualization, level 1, was defined as an event in which a new disease candidate is discovered and officially reported. The concept formulation phase (level 2) was defined as the formal proposal of a new disease concept based on accumulated knowledge through case series. The transition from level 1 to level 2 of the DRL was indicated by the following statement of autosomal dominant TRPV4 disorders as an example: “We propose that AD brachyolmia lies at the mildest end of this spectrum and, since all cases described with this diagnosis and TRPV4 mutations display metaphyseal changes, we suggest that it is not a distinct entity but represents the mildest phenotypic expression of SMDK [39].”Considering the extensive role of patient organizations required in patient education, research and clinical trial support [40, 41], they could be established as early as level 2, depending on the number of patients and the characteristics of the disease. Levels 3–4 were the phases of advancements in the basic and applied research concerning the target disease. At level 5, the concept of the disease was generally complete, with formal diagnostic criteria and/or standard practice for treatment in clinical practice guidelines using the evidence accumulated up to level 4. Diagnostic methods, treatments, and mechanisms for diseases included up to level 4 of the DRL were components in clinical practice guidelines [42–44]. For example, in the first clinical practice guideline for intravenous immunoglobulin of neuromuscular diseases including several RDs, the transition from evidence accumulation to guideline development, i.e., from level 4 to level 5, was marked by stating the following: “The list of diseases in which IVIg has proven efficacious has now expanded to cover various demyelinating neuropathies, neuromuscular transmission defects, and inflammatory myopathies.”, “Information regarding the diseases in which IVIg is clinically indicated or effective, as well as data on how it works and how best to administer it, can be used by practicing neurologists to make judicious decisions in their clinical practice [45].”Level 6 was defined as the stage of screening and identification of therapeutic drug candidates using disease models. Levels 7–9 comprised the preclinical study, clinical development, and regulatory approval phases of the drugs through clinical trials. RDs often do not follow the three individual steps of phases 1, 2, and 3 in clinical trials due to their small number of patients, increasing instances of development of new modalities such as gene therapy, and the use of various fast-track approaches [5, 46, 47]. Thus, we have included phase 1, 2, and 3 clinical trials into one level as level 8. The Orphan Drug Designation (ODD) is obtained at this level because clinical trial data on efficacy and safety are generally required for designation as an orphan medicine [3, 48]. An important contribution of patient organizations at level 8 is the provision of patient-oriented information for clinical trials [41]. Level 9 was divided into three subsections according to the degree of treatment effect (i.e., symptomatic, disease-modifying, or curative) of the approved drug on the disease. There are fewer steps at level 6, 7 and 8 in DRL, but if subdivision is needed for product-specific grading, the BARDA TRL for Medical Countermeasure Products may be used. The relationship between our proposed DRL and TRL is shown in Fig. 1. The figure of the relationship was developed with reference to the CRI [30].

DRL: Disease Readiness Levels, iPS: induced pluripotent stem, ICD: International Statistical Classification of Diseases and Related Health Problems, PPI; Patient and Public Involvement

### Case selection

To evaluate the validity of the proposed framework as a pilot study, we selected the following RDs with low, medium, and high levels of research progress and understanding: muscular dystrophy, fibrodysplasia ossificans progressive (FOP), Tangier disease (TGD), and idiopathic peripheral pulmonary artery stenosis (PPAS). Except for idiopathic PPAS, for which the concept of the disease has recently been proposed [49], the other three diseases are rare and designated as “intractable diseases” in Japan [50]. Muscular dystrophy is a fatal genetic disorder characterized by progressive weakness and degeneration of the skeletal muscles. In recent years, advancements in the development of therapeutic drugs, especially for Duchenne muscular dystrophy (DMD), have been made, with ongoing research focusing on innovative approaches to address the underlying genetic factors [51, 52]. FOP is an ultra-rare disease characterized by congenital bilateral hallux valgus and early onset heterotopic ossification [53]. Although no disease-specific treatment exists for FOP, applied research has focused on the use of induced pluripotent stem (iPS) cells as a compound screening system [54]. Patients with TGD and heterozygous carriers of *ABCA1* variant are characterized by severe deficiency or absence of high-density lipoprotein in the circulation, resulting in the accumulation of cholesteryl esters [55]. A certain level of consensus exists on the common diagnostic criteria and disease definitions for these cases in different countries and regions. The following result section includes each analysis result, along with a concise disease definition, though some overlap with the information presented in this section.

## Results

**Fig. 2:**
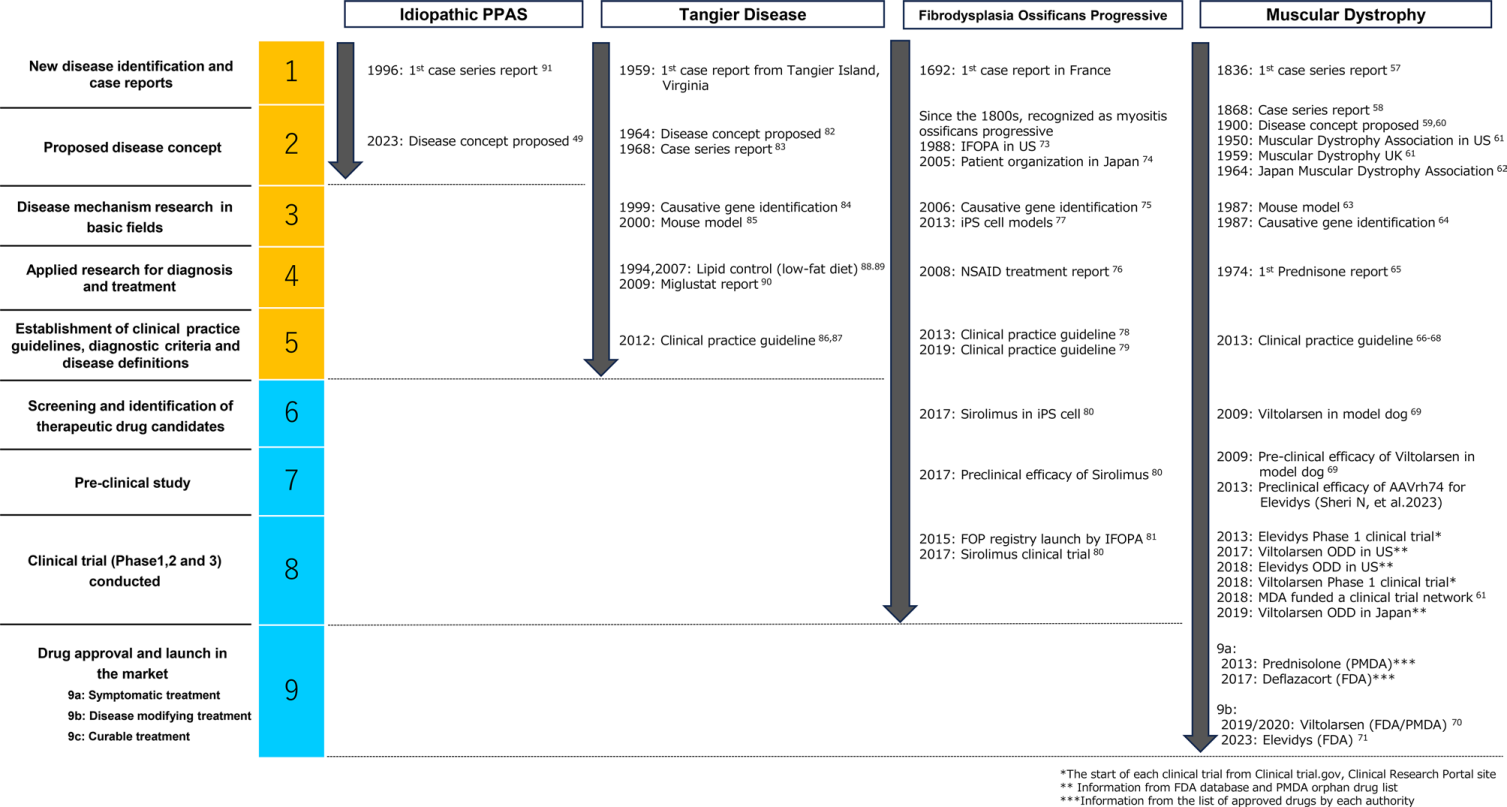
Determination of the disease readiness levels in the cases. Grading by DRL is performed for idiopathic ppas, Tangier disease, fibrodysplasia ossificans progressive and muscular dystrophy. DRL, disease readiness Levels; odd, orphan Drug Designation; ppas, peripheral pulmonary artery stenosis; fda, food and Drug Administration; IFOPA, International fibrodysplasia ossificans progressive Association; iPS, induced pluripotent stem; PMDA, pharmaceuticals and medical devices agency

The DRL framework comprehensively describes the identification of the target disease, progress of research, establishment of the disease definition and concept, and launch of the therapeutic strategy. The DRL assesses the entire disease, including the pre-technology readiness phase, i.e., prior to the product development assessed by the TRL. Grading of the four cases was performed using the DRL framework (Fig. 2).

### Muscular dystrophy

Muscular dystrophy comprises a group of genetic disorders characterized by progressive muscle weakness and wasting [56]. Muscular dystrophy could be assigned to level 9, with research and development focusing on DMD, the most common type of muscular dystrophy. Small-molecule drugs, as well as exon-skipping gene therapy have been launched. The first case of DMD was reported in Italy in 1836 [57], and a case series and the disease concept were published by Duchenne in 1868 [58]. Multiple cases of muscular dystrophy and autopsies have been reported since 1900 [59, 60]. Patient organizations for muscular dystrophy were established during this period [61, 62]. A typical example of clinical trial acceleration activities by patient organizations is the investment of Muscular Dystrophy Association in a clinical trial network [61]. Following the establishment of an animal model [63], *DMD* gene cloning [64], and the first case demonstrating the effectiveness of prednisone [65], clinical practice guidelines, including non-pharmacological treatments, were established [66–68]. Additionally, viltolarsen, approved for the treatment of DMD, was granted ODD by the FDA in 2017, the timing of level 8, according to the Orphan Drug Product designation database. Despite limited targeted genetic mutations, treatments using exon-skipping methods have been approved and marketed [52, 69–71].

### Fibrodysplasia ossificans progressive

FOP is an extremely rare autosomal dominant disorder characterized by congenital malformations of the great toes and progressive heterotopic ossification [72]. FOP was placed at level 8. The first case of FOP, which was reported in France in 1692, was initially recognized as myositis ossificans progressiva in the 1800s. Patient organizations for FOP were established in 1988 and 2005 [73, 74]. The causative gene, *ACVR1*, was identified in 2006 [75], and the symptomatic treatments were reported [76]. iPS models were constructed in 2013 [77]. Clinical practice guidelines, including the use of non-steroidal anti-inflammatory drug treatment and non-pharmacologic management, have been established since 2013 [78, 79]. Although no approved therapies specific to FOP exist, a clinical trial is being conducted using sirolimus, which was screened using iPS models [80]. Furthermore, international patient organizations in FOP are providing clinical trial information and operating a patient registry to accelerate research and development [81].

### Tangier disease

TGD is a rare genetic disorder characterized by severe deficiency or absence of High-density lipoprotein in the circulation resulting in tissue accumulation of cholesteryl esters throughout the body, particularly in the reticuloendothelial system [55]. TGD was placed at level 5. The first case of TGD was reported in Tangier Island, Virginia, in 1959. The disease concept was proposed based on a case series published in 1968 [82, 83]. In 1999, the causative gene, *ABCA1*, was identified, and a mouse model was developed in 2000 [84, 85]. Clinical practice guidelines have been developed in several countries since 2012 [86, 87]. Although potential efficacy of low-fat diet [88, 89] and miglustat, an existing drug for diseases other than TGD [90], has been reported, specific therapeutic development for TGD was not attained at the time of level determination.

### Idiopathic peripheral pulmonary artery stenosis

Idiopathic PPAS is an idiopathic adult-onset disease characterized by stenosis of the pulmonary artery from the trunk to the peripheral arteries [49]. Idiopathic PPAS was placed at level 2. Originally recognized as secondary PPAS, a case series of adult PPAS was reported in 1996 [91], and the disease concept of idiopathic PPAS was first established in 2023 [49]. Both basic and applied research is currently in the initial phase as the diagnostic criteria for clearly distinguishing idiopathic PPAS from other similar diseases (e.g., pulmonary arterial hypertension and chronic thromboembolic pulmonary hypertension) and clinical practice guidelines have not yet been developed, and the disease name has not yet been listed in the latest ICD codes.

## Discussion

We developed a DRL based on the NASA-originated TRL, that allows for a grading of the understanding of the disease, rather than the product, to be the target of the assess. The DRL was also validated using several rare diseases as cases. Based on the results of a pilot study of four case diseases, the proposed DRL has three distinct advantages. First, this allows the comparison of the level of understanding and research progress across multiple diseases. The DRL focused on the phases prior to the clinical trials for new drug applications, such as the establishment of the disease concept, clinical practice guidelines, and the research phase. The stepwise assessment of the disease is a novel approach that includes the research phase prior to the technology readiness assessed by the TRL. Second, DRL is expected to provide a relative assessment of the level of understanding of RDs and will help not only the pharmaceutical industry but also other stakeholders, such as the government and patient groups, to compare different diseases and consider appropriate interventions. Finally, DRL can also visualize the progress of medical science in the target disease.

### Comparison of the DRL assessment phase with TRL derivatives

Previous TRL derivatives in the medical field, such as animal drugs (Drugs for food animals, Drugs for companion animals, and Veterinary vaccines), RRL and BARDA TRLs have assessed the technology maturity of the product unit [26, 28, 29]. When the RRL and BARDA TRLs frameworks were used for RDs, DMD was placed at level 9, and FOP was placed at level 8, which were both similar to that of the DRL. The assessment of level of completeness remains the same for developing therapeutic strategies; however, the resolution of the process varies considerably. Although the RRL focuses on the process of new drug development, DRL highlights the process of disease identification, elucidation of the cause of the disease, and development of clinical practice guidelines, including the availability of existing drugs. The new drug development process is organized at beyond level 6 to ensure progress on the DRL. The DRL is a linear framework for assessing progress in disease research and is appropriate for evaluating status change. In practice, the progress of new clinical practice guideline establishment (DRL level 5) and product development (DRL levels 6–9) is iterative, interspersed with clinical practice guideline updates, resulting in further maturation of the level 9 subsections 9a through 9c. A spider graph analysis with identified factors, as previously reported [92], may be effective for drug and portfolio-specific evaluations in DRL level 9. In contrast to DMD and FOP, TGD and idiopathic PPAS were both placed at level 1 in the RRL or BARDA TRLs grading; however, a clear difference was present between the two diseases in terms of research progress and disease understanding in the DRL. The DRL grading confirmed the placement of TGD at level 5, with established clinical practice guidelines, treatment strategies, and tools. The DRL grading of idiopathic PPAS was at level 2, which indicates the development of disease concept and no intervention measures or clinical practice guidelines. The results of the comparison between DRL and existing TRL derivatives in the medical field suggest that DRL, by placing the establishment of clinical guidelines at level 5, can extend the assessment phase to before product development and clarify the differences in the understanding of RDs and medical intervention policies, as well as the differences in the progress of research before product development.

### UMNs assessment and DRL

Identification of UMNs is an important factor in RD research. While UMNs have been explained by the availability of therapeutic drugs for development [93], stakeholder perspectives, including the adequacy of alternative treatments, disease burden, population size [9], and the balance between drug contribution and treatment satisfaction through survey scores [94], a universal definition or method for quantifying UMNs has not been established. Although the DRL does not directly quantify UMNs, diseases with low DRL levels in RDs indirectly correspond to high UMNs owing to lack of access to accurate diagnostic methods and both drug and non-drug treatments for alleviating symptoms and disease burden. Since UMNs, which are difficult to quantify, can now be compared across multiple diseases using DRL, UMNs can be used as an indicator to determine the focus of the investments of all stakeholders. As UMNs vary based on the stakeholder’s perspective [9], DRLs can be used by multiple stakeholders as a common tool for assessing UMNs. Further research combining and validating DRL and other indicators, such as surveys and multi-stakeholder consensus working groups for the assessment of UMNs, is warranted.

### Influence of the disease characteristics on the DRL assessment results

Most RDs have a genetic etiology [95]. Genetic diseases are identified based on the clinical symptoms and family history, and the disease mechanism is studied by variant analysis of the causative genes. Therefore, various animal models have been created using genetic engineering. DMD selected for determining the clinical evidence in this study is an inherited muscle disease. Therefore, while DMD met each definition of the DRL, it attained the level of the launch of disease-modifying treatment, that is, level 9b as shown in Fig. 2. However, pharmaceutical treatment regimens have been established for certain diseases, such as diffuse panbronchiolitis [96], without elucidating the therapeutic mechanism. The understanding of the disease may vary based on the scientific era in which the disease was discovered and identified owing to differences in the available research and treatment modalities. Cases that undergo such atypical research steps may be incompletely and heterogeneously evaluated using DRL in terms of disease understanding and research progress. As RDs also comprise non-genetic diseases, further studies including a larger number of diseases are warranted to demonstrate the validity of the evaluation by DRL. In addition, the process of understanding a disease may be different for common diseases, which have been studied in a larger number of patients by many researchers compared to RDs. High medical needs, defined as the lack of alternative therapies, were more frequently claimed in orphan drug applications; however, the criteria for regulatory review and approval decisions for orphan and non-orphan drugs did not differ [97]. Progress in the disease understanding process may vary in the DRL between RDs and common diseases. Further research is warranted to determine the need for a modified DRL for common diseases and the differences in the interventions required for rare and common diseases.

### Resource allocation to RDs

The current policy on orphan drugs encourages their development by providing incentives for RDs with low prevalence [3]. However, no clear guidelines exist as to which of the many RDs should be prioritized for R&D investment support. Verification of the appropriateness of the allocation of research funds by the administration to RDs was not sufficient [32]. The prevalence of a disease correlates with the level of research progress, and the number of studies was high for RDs with a relatively high prevalence [98]. However, some argue that policymakers and government funding agencies should prioritize research investments based on the severity of a disease rather than its prevalence [99]. Owing to the difficulty in objectively comparing the severity of multiple RDs due to varying pathologies and impact on patients, organizing the research progress and understanding multiple diseases in a comparable manner using our proposed DRL grading as a preliminary step could aid in the allocation of investments. The Rare Disease Moonshot identifies the fragmentation of rare disease research [37], and the DRL can contribute to this challenge by organizing information on the research progress in a unified framework. Once several RDs have been ranked by the DRL, information on the diseases needing interventions can be provided to stakeholders, such as policy makers, government funding agencies, industry, and patient groups. For funding requesters, grading by DRL would be expected to provide a basis for funding requests and milestone achievement. Industry R&D investment in rare diseases is inadequate for pediatric onset diseases and for diseases with particularly small patient populations, and tends to focus on diseases with adult onset, larger market size, and low risk of immediate death [100]. Visualizing R&D progress across multiple rare diseases through DRL may also help correct this imbalance in industry investment. Our study was a preliminary grading study based on the proposed DRL. Therefore, further research in the form of an empirical study is warranted to increase the number of target diseases and examine actual interferences and their effects to determine the optimal intervention for each DRL level by every stakeholder.

### Contribution by patient organizations

Patient advocacy is becoming essential and important part of orphan drug development, regardless of country or area [101, 102]. In the proposed DRL, we have described the representative activities associated with patient organizations, such as the establishment of patient organizations and Patient and Public Involvement (PPI) in the definition of level 2 and level 8 in Table 1, respectively. The activities of patient organizations go beyond DRL and have an impact that extends beyond therapeutic drug development to disease care. Since a disease concept must be established before a patient organization can be established, the definition at level 2 includes the starting point for patient organization activities. Involvement in clinical trials at level 8 has been incorporated in the definition at level 8 because it is expected to be the most important contribution of patient organizations as PPI. However, the scope of the contribution of patient organizations is potentially broader; for example, there are representative five activities that muscular dystrophy patient organizations can contribute to, such as establishing national registries, identifying and address barriers in clinical trials, collaborating with industries to integrate patient needs, working on disease-specific regulatory guidance and integrating market access insights [61]. The first of these activities, establishing national registries, can correspond not only to DRL level 8, but also to Levels 4 through 9 and beyond. Identifying and addressing barriers to clinical trials is at level 8, collaborating with industries to integrate patient needs is at levels 6–9, working on disease-specific regulatory guidance is at levels 7–9, and integrating market access insights can be related to levels 8 and 9. Thus, while the DRL framework can be extended to the activities of patient organizations, it may not be possible to assess all activities because all information on patient organizations is often not publicly available, so it is preferable for each patient organization to use the DRL framework to assess its own activities.

### Limitations and future research

Although this study presents a novel approach, it has some limitations. First, the number of RDs assessed as cases in this study was small. Further research is warranted on diseases designated as orphan diseases in many countries and intractable diseases in Japan (341 diseases as of April 2024) [103]). The definitions at each level of the DRL should be updated to allow the understanding grade of different diseases. Second, when collecting disease-specific information for leveling, differences in the quality of the information prevent accurate tiering, particularly for lower-level diseases. Lower-level diseases with relatively less advanced research may have more publications in non-English languages, which may make accessing data for base leveling challenging. Thus, room for interpretation and ambiguity may exist in the assessments made by implementers when determining the levels using the DRL framework. Conversely, differences in level determination may exist among implementers even in diseases with high levels and advanced research, with very large amount of data for level determination or multiple discourses for which consensus has not been reached. Third, DRL cannot optimally assess RDs that are primarily treated with non-pharmacological therapies because we proposed DRL focused on understanding drug treatment. For RDs treated with non-pharmacological therapies, the DRL remains at level 5 even though the unmet medical need has been addressed and medical science has progressed. If DRL is to be expanded to non-drug therapies, such as medical devices, it will be necessary to generalize the DRL definition, e.g., DRL level 6 to “prototyping” and DRL level 9 to “regulatory approval”. Fourth, our DRL focuses on the stepwise-process of the first drug to treat the RD (“first in disease”). The DRL framework prioritizes tracing the history of “first in disease” at each level, but at level 6 and above, it becomes cumbersome to describe the history of multiple drugs. A modified framework and methodology are needed to evaluate the development of multiple types of interventions in a given rare disease. Finally, the level determination in this study was set mainly in terms of the level of progress in R&D. In addition to the level of research progress, stakeholder mapping is also important for a comprehensive understanding the disease landscape. Stakeholder readiness should be measured using other tools, such as START [19].

## Conclusions

This study designed a comprehensive framework spanning disease discovery to therapeutic drug development by introducing DRL, which focused on the disease rather than the product for assessment, and analyzed four RDs with different degrees of disease understanding and therapeutic drug development. Our findings suggest that the grading of the R&D progress by DRL may be used to compare RDs. Additionally, the DRL framework may be used to analyze both the level of understanding of rare disease and the progress of the product R&D and serve as a reference index for the allocation of R&D resources for multiple stakeholders in medical field.

## Data Availability

Data sharing is not applicable to this article as no datasets were generated or analyzed during the current study.

## Abbreviations

ABCA1: ATP binding cassette subfamily A member 1
ACVR1: Activin A receptor type 1
BARDA: The Biomedical Advanced Research and Development Authority
CRI: Commercial Readiness Index
DMD: Duchenne muscular dystrophy
DRL: Disease Readiness Levels
EMA: European Medicines Agency
FDA: Food and Drug Administration
FOP: Fibrodysplasia ossificans progressive
GLP: Good Laboratory Practice
GMP: Good Manufacturing Practice
ICD: International Statistical Classification of Diseases and Related Health Problems
IND: Investigational new drug
iPS: induced pluripotent stem
NASA: National Aeronautics and Space Administration
ODD: Orphan Drug Designation
PD: pharmacodynamics
PK: pharmacokinetics
PMDA: Pharmaceuticals and Medical Devices Agency
PPAS: Peripheral pulmonary artery stenosis
PPI: Patient and Public Involvement
RDs: Rare diseases
R&D: Research and development
RRL: Regulatory Readiness Levels
TGD: Tangier disease
TRL: Technology Readiness Levels
TRPV4: Transient receptor potential cation channel subfamily V member 4
UMNs: Unmet medical needs
